# Identification and experimental validation of ferroptosis-related gene lactotransferrin in age-related hearing loss

**DOI:** 10.3389/fnagi.2024.1309115

**Published:** 2024-01-11

**Authors:** Chaojun Zeng, Xi Gu, Yuqing Chen, Yanchun Lin, Junying Chen, Zhifeng Chen, Chenyu Chen, Guangnan Yao, Chang Lin

**Affiliations:** ^1^Department of Otorhinolaryngology Head and Neck Surgery, The First Affiliated Hospital, Fujian Medical University, Fuzhou, China; ^2^Department of Otorhinolaryngology Head and Neck Surgery, National Regional Medical Center, Binhai Campus of the First Affiliated Hospital, Fujian Medical University, Fuzhou, China; ^3^Fujian Institute of Otolaryngology, The First Affiliated Hospital, Fujian Medical University, Fuzhou, China; ^4^Central Laboratory, Key Laboratory of Radiation Biology of Fujian Higher Education Institutions, The First Affiliated Hospital, Fujian Medical University, Fuzhou, China; ^5^Central Laboratory, Key Laboratory of Radiation Biology of Fujian Higher Education Institutions, National Regional Medical Center, Binhai Campus of the First Affiliated Hospital, Fujian Medical University, Fuzhou, China

**Keywords:** age-related hearing loss, ferroptosis, lactotransferrin, hub gene, mechanism

## Abstract

**Objective:**

To reveal the relationship between ARHL and ferroptosis and screen ferroptosis-related genes (FRGs) in ARHL.

**Methods:**

Bioinformatics were used to analyze the hub genes and molecular mechanism of ferroptosis in the aging cochleae. Senescence β-galactosidase staining, iron content detection, and micro malondialdehyde (MDA) assay kits were used to measure β-galactosidase activity, and expression of Fe^2+^ and MDA, respectively. Fluorescence microscope was used for immunofluorescence assay of hub genes. Western blot was used to verify the expression of hub genes in HEI-OC1 cells, cochlear explants, and cochleae of C57BL/6J mice. Data were expressed as mean ± SD of at least three independent experiments.

**Results:**

The analysis of bioinformatics confirmed that lactotransferrin (LTF) is the hub gene and CEBPA-miR-130b-LTF network is the molecular mechanism for cochlear ferroptosis. Compared with the control group, the experiments proved that the indicators of ferroptosis, including Fe^2+^, MDA, and LTF were differentially expressed in aging HEI-OC1 cells, aging cochlear explants, and aging cochleae.

**Conclusion:**

These results demonstrate that ferroptosis plays an important role in ARHL, and LTF is a potential therapeutic target for ARHL via regulating cochlear ferroptosis.

## Introduction

Age-related hearing loss (ARHL), also known as presbycusis, is characterized by bilateral symmetrical sensorineural hearing loss mainly at high-frequency. Currently, ARHL is only treated with cochlear implants or hearing aids, and the number of patients will be over 500 million by 2025 (World Health Organization, 2018). Moreover, ARHL is one of the top five level three causes of years lived with disability (GBD 2019 Ageing Collaborators, 2022) and increases the risk of depression, cognitive decline, and injuries from falling (Vaisbuch and Santa Maria, 2018). Hair cell loss, stria vascularis atrophy, and spiral ganglion neuron degeneration are the main causes of ARHL (Keithley, 2020). However, the exact mechanisms of ARHL are still unknown which mainly focuses on apoptosis (Wu et al., 2022) and autophagy (Cho et al., 2022).

Ferroptosis, which is caused by iron overload and lipid peroxidation, was officially classified as a novel form of regulated cell death in 2018 (Galluzzi et al., 2018; Hirschhorn and Stockwell, 2019). Ferroptosis has become the emerging mechanism and therapeutic target for neurodegenerative disease (Wang et al., 2022) and is involved in aging in numerous organs (Stockwell, 2022). Recently, ferroptosis was confirmed to be related to neurodegeneration of the auditory cortex in aging rats (Chen et al., 2020), and inhibition of ferroptosis reduced hair cell loss induced by neomycin or cisplatin (Hu et al., 2020; Zheng et al., 2020). Nevertheless, it has not been reported whether hair cell damage is related to ferroptosis in ARHL. Meanwhile, changes in miRNA expression may lead to presbycusis by inhibiting the development of the inner ear and impairing its homeostasis (Chen et al., 2019; Yoshimura et al., 2019). Several studies have concentrated on the role of miRNAs and ferroptosis in various diseases, including Alzheimer’s disease (Tan et al., 2023), ankylosing spondylitis (Zong et al., 2022), and tumors (Dai et al., 2022). Nevertheless, it has not been investigated whether ferroptosis and related miRNA regulatory networks are involved in ARHL.

**Figure 1:**
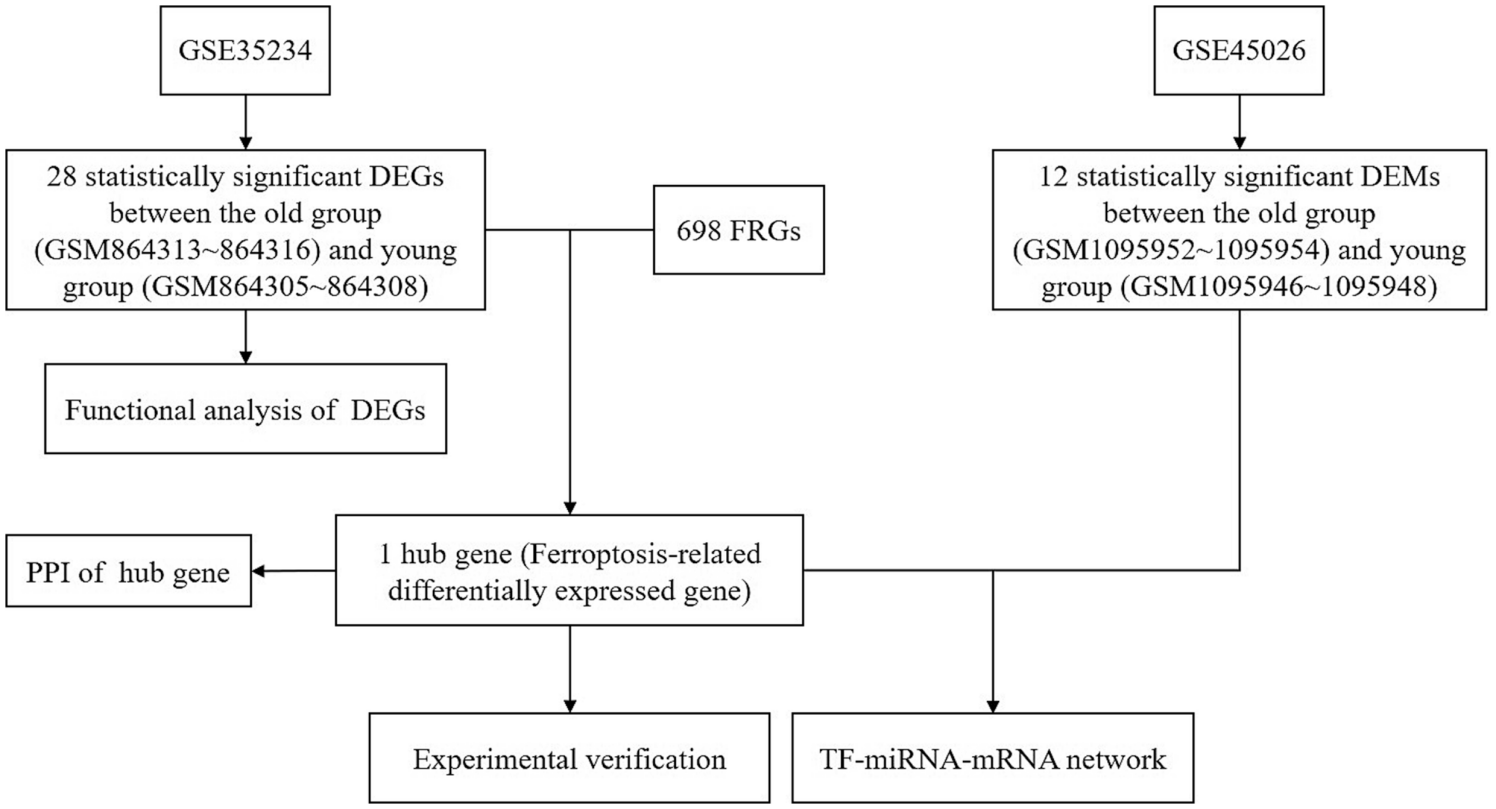
The protocol of this study. DEGs, differentially expressed genes; DEMs, differentially expressed miRNAs; FRGs, ferroptosis-related genes; PPI, protein–protein interaction.

The aim of this study was to explore the relationship between ferroptosis and ARHL, construct related regulatory networks using bioinformatics, and verify potential therapeutic target genes for ARHL *in vivo* and *in vitro* (The flow chart was shown in Figure 1).

## Materials and methods

### Identification of differentially expressed genes (DEGs) and differentially expressed miRNAs (DEMs)

Two datasets of GSE35234 and GSE45026 were both from the gene expression omnibus (GEO) database.^1^ Cochlear tissues from C57BL/6J mice of different ages were selected for analysis. To identify the DEGs, four samples (GSM864313, GSM864314, GSM864315, and GSM864316) were defined as the old group, and another four samples (GSM864305, GSM864306, GSM864307, and GSM864308) were defined as the young group in the GSE35234 dataset. To identify the DEMs, three samples (GSM1095952, GSM1095953, and GSM1095954) were defined as the old group, and another three samples (GSM1095946, GSM1095947, and GSM1095948) were defined as the young group in the GSE45026 dataset. |log2(FC)| > 1 and adjusted *p*-value <0.05 were considered statistically significant.

The GEO2R online tool^2^ was used to identify DEGs and DEMs.

### Functional analysis of DEGs

The WebGestalt database^3^ was used to analyze the biological functions of the DEGs.

### Identification of ferroptosis-related genes (FRGs)

The GeneCards database^4^ was used to search FRGs. The Venny online tool^5^ was used to cross-analyze overlapping genes and identify hub genes.

### Construction of the TF-miRNA-mRNA network

The STRING database^6^ was used to analyze the protein–protein interaction (PPI) network of hub genes with the minimum required interaction score set to 0.4 (medium confidence). Then, the PPI network was visualized by Cytoscape (version 3.6.1) software platform. The TargetScan database^7^ was used to predict the target genes of miRNAs. The GeneCards database was used to verify the homology of hub genes in both mice and humans, and the miRBase database^8^ was used to match the miRNAs of the two species. The miRNet,^9^ TransmiR,^10^ and Cistrome DB databases^11^ were used to predict transcription factors (TFs), then the overlapping TFs were identified by cross-analyzation.

### Cell line

The HEI-OC1 cell line was obtained from Professor Wenyan Li (He et al., 2020). HEI-OC1 auditory cells were cultured in high-glucose DMEM medium (Meilunbio, China) containing 10% fetal bovine serum (FBS, Gibco, Mexico) and 50 μg/mL ampicillin (Sangon Biotech, China) for 12 h at 33°C with 10% CO_2_. Then, different concentrations (10–20–30-40 mg/mL) of D-gal (Meilunbio, China) or 5 μm Liproxstatin-1 (MedChemExpress, USA) were applied to the culture medium, and an equal volume of PBS was added to the control group. Finally, cells were cultured for another 48 h before subsequent experiments.

### Cochlear explants

The cochleae of postnatal day 3 (P3) C57BL/6J mice were dissected in transparent Hank’s balanced salt solution (Meilunbio, China). After removing the lateral wall and spiral ligament, the basilar membrane was laid flat on a crawling sheet soaked with polylysine (Meilunbio, China). These cochlear tissues were cultured in DMEM/F-12 medium (Meilunbio, China) containing 10% FBS and 1% penicillin G (Sangon Biotech, China) for 12 h at 37°C with 5% CO_2_. Then different concentrations (10-20-30-40 mg/mL) of D-gal were applied to the culture medium, and an equal volume of PBS was added to the control group. Finally, cochlear tissues were cultured for another 48 h before subsequent experiments.

### Animals

Twelve male C57BL/6J mice were accommodated in the Laboratory Animal Center of Fujian Medical University. Mice were divided into two groups, with six of them in the old group (10-month-old) and the other six in the young group (2-month-old). The animal study protocol was approved by the Animal Ethics Committee of Fujian Medical University (approval No. IACUC FJMU 2022-0623).

### Measurement of auditory brainstem response (ABR) threshold

The ABR equipment (Neuro-Audio, Russia) was accurately calibrated by the National Institute of Metrology (Report No.: LSsx2022-00028), and Neuro-Audio software (Version 1.0.105.1, Russia) was used to analyze the ABR threshold in mice. Briefly, mice were sedated with 0.1 mL/kg xylazine hydrochloride (Sangon Biotech, China) and under anesthesia with 1 mL/kg pentobarbital sodium (Merck, Germany). According to operating instructions, three needle electrodes were inserted at the vertex of the midline, and posterior to bilateral ears. Auditory stimulation was performed at 10, 20, and 30 kHz, with decreasing sound intensity processing from 100 dB to 0 dB, with 5 dB intervals to identify the threshold.

### Measurement of senescence

A senescence β-galactosidase staining kit (Beyotime, China) was used to measure the extent of senescence according to the manufacturer’s instructions. Briefly, the treated HEI-OC1 cells or cochlear tissues were fixed with fixing solution for 15 min. After washing with PBS, they were stained with staining solution at 37°C without CO_2_ overnight. Stained HEI-OC1 cells or cochlear tissues were observed via optical microscope (Olympus, CX41, Japan), and the staining field was taken randomly to calculate the number of senescent cells per 100 cells.

### Measurement of Fe^2+^

An iron content detection kit (Solarbio, China) was used to measure the content of Fe^2+^ according to the manufacturer’s instructions. Briefly, the treated HEI-OC1 cells or cochlear tissues were lysed with extract and centrifuged at 4,000 × *g* for 10 min at 4°C. The supernatant was collected and boiled for 5 min after being mixed with detection reagent. Then chloroform was added to the mixture and centrifuged at 10,000 × rpm for 10 min at room temperature. The newly collected supernatant was added to a 96-well plate, and then the absorbance of the samples at 520 nm was recorded using a SpectraMax i3x (Molecular Devices, USA). The protein concentration of the samples was quantified by a BCA kit (Meilunbio, China). Relative Fe^2+^ level was expressed as μg/mg protein.

### Measurement of lipid peroxide

Malondialdehyde (MDA), an end product of lipid peroxidation, was measured using a micro malondialdehyde (MDA) assay kit (Solarbio, China) according to the manufacturer’s instructions. Briefly, the treated HEI-OC1 cells or cochlear tissues were lysed with extract and centrifuged at 8,000 × *g* for 10 min at 4°C. The supernatant was collected and boiled for 60 min after being mixed with detection reagent. New supernatant was collected after the mixture was centrifuged at 10,000 × *g* for 10 min at room temperature and added to a 96-well plate. Then the absorbance of the samples at 532 nm and 600 nm was recorded using a SpectraMax i3x. Relative MDA level was expressed as nmol/mg protein.

### Western blot analysis

The samples were lysed with RIPA lysis buffer (Meilunbio, China) for protein extraction. The supernatants were collected for protein analysis after the lysates were centrifuged at 14,000 × *g* for 15 min at 4°C. Protein samples were separated via 10% SDS-PAGE (Meilunbio, China) and transferred to PVDF membranes (0.2 μm, Millipore, Bedford, MA, USA). After probing with rabbit anti-LTF (1:500, BOSTER, China) and anti-β-actin (1:1000, Proteintech, China) overnight at 4°C, the membranes were incubated with goat anti-rabbit IgG (10000, Immunoway, USA) for 1 h. Protein bands were detected using an ECL kit (Meilunbio, China). Densitometry was performed with ImageJ software.

### Immunofluorescence staining and analysis

Harvested HEI-OC1 cells were used for immunofluorescence assay. Briefly, cells were fixed with 4% paraformaldehyde for 20 min, permeabilized with 0.5% Triton X-100 for 15 min, and blocked with 10% BSA for 10 min. After probing with rabbit anti-LTF (1:50, BOSTER, China) at 4°C overnight, the cells were incubated with goat anti-rabbit IgG (H&L) – Alexa Fluor 488 (1:100, Immunoway, USA) for 1 h. Nuclei were visualized via DAPI (Meilunbio, China). Images were detected using a fluorescence microscope (Olympus, BX3, Japan).

### Statistical analysis

Data were expressed as mean ± SD of at least three independent experiments. Unpaired two-tailed Student’s t-test was used to compare the means between two groups, and one-way or two-way ANOVA was used to analyze the data between three or more groups after Dunnett correction in GraphPad Prism 8.0 program (San Diego, CA, USA). NS = not significant, *p* < 0.05 was considered statistically significant (All data were shown in Supplementary material).

## Results

### Differentially expressed genes (DEGs) in ARHL

**Figure 2:**
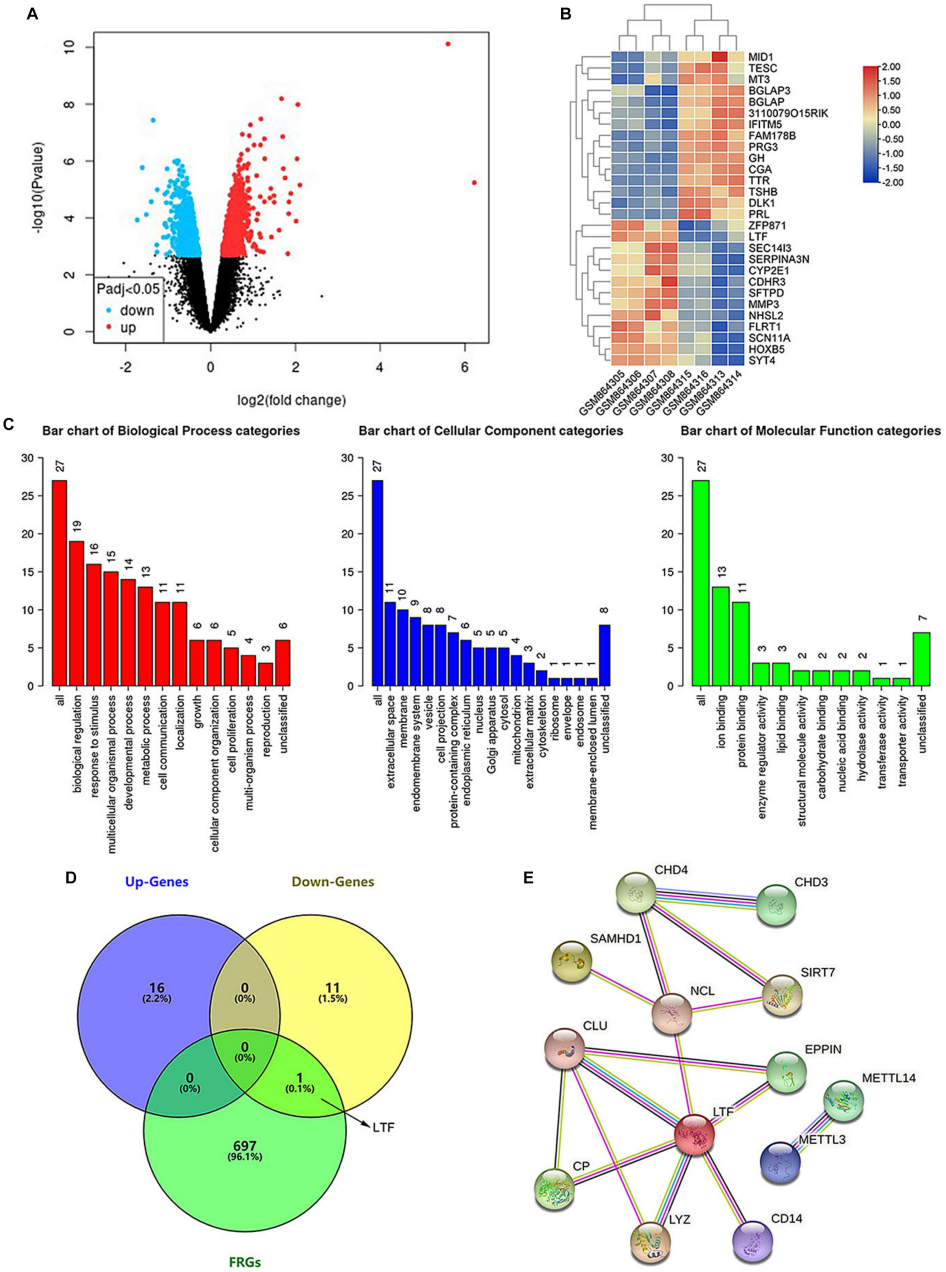
LTF, a ferroptosis-related gene, is identified in aging cochleae. **(A)** The volcano diagram of GSE35234 shows 43385 DEGs between the old group (GSM864313~864316) and young group (GSM864305~864308). **(B)** The heatmap shows 28 statistically significant DEGs. **(C)** Functional analysis of the statistically significant DEGs. **(D)** LTF, the hub gene, is obtained from the intersection of Up-Gens, Down-Gens (based on the statistically significant DEGs), and FRGs. **(E)** The PPI network shows that 12 proteins are interacting with LTF.

43385 DEGs between the old group (GSM864313~864316) and young group (GSM864305~864308) in the GSE35234 dataset (Data were shown in Supplementary material) were identified with GEO2R (Figure 2A). 28 of them were further identified by the indicators: |log2(FC)| > 1 and adjusted *p*-value <0.05 (Figure 2B). The biological functions of the identified DEGs were analyzed using the WebGestalt database (Figure 2C). One gene was unable to be found in Entrez Gene ID, so the function of 27 DEGs was analyzed. DEGs controlled biological regulation, response to stimulus, multicellular organismal processes, developmental processes, and metabolic processes of the biological processes. DEGs had cellular components that were mainly associated with extracellular space and membrane bilayers. The molecular functions were associated with ion binding, protein binding enzyme regulator activity, and lipid binding. The results were consistent with the characteristics of ferroptosis, i.e., the disorder of intracellular iron metabolism, which causes lipid peroxidation and leads to cytomembrane rupture.

### FRGs cause ARHL

A total of 698 FRGs were searched using the GeneCards database (Data were shown in Supplementary material). LTF, the only overlapping gene, was identified by cross-analyzing between FRGs and DEGs (Figure 2D). The functions of LTF involve regulating iron homeostasis, antioxidation, anti-inflammation, and anticancer (Artym et al., 2021; Bukowska-Ośko et al., 2022; Kowalczyk et al., 2022). LTF, as a ferroptosis-related gene, was related to the differential expression gene of aging cochleae. Therefore, we hypothesized that LTF is a candidate gene for regulating cochlear ferroptosis and was validated in subsequent experiments.

### Construction network of protein-protein interaction (PPI)

PPI is made up of functionally similar proteins that is critical to understanding biological processes (Wanker et al., 2019). PPI demonstrates the importance of hub genes and is a valuable tool for identifying novel protein functions (Revathi Paramasivam et al., 2021). The STRING database contains information on more than 5,000 species, 20 million proteins, and 3 billion interactions. The PPI constructed by the STRING database in this work includes the hub gene of LTF and 12 matching interacting genes (Figure 2E). Among these are SIRT7 (Li X. T. et al., 2022), METTL14 (Zhuang et al., 2023), METTL3 (Lin et al., 2022), CP (Shang et al., 2020), and CD14 (Hu et al., 2021), which have also been confirmed to be associated with ferroptosis. It means that LTF, as a hub gene, is closely related to ferroptosis.

### Differentially expressed miRNAs (DEMs) in ARHL

**Figure 3:**
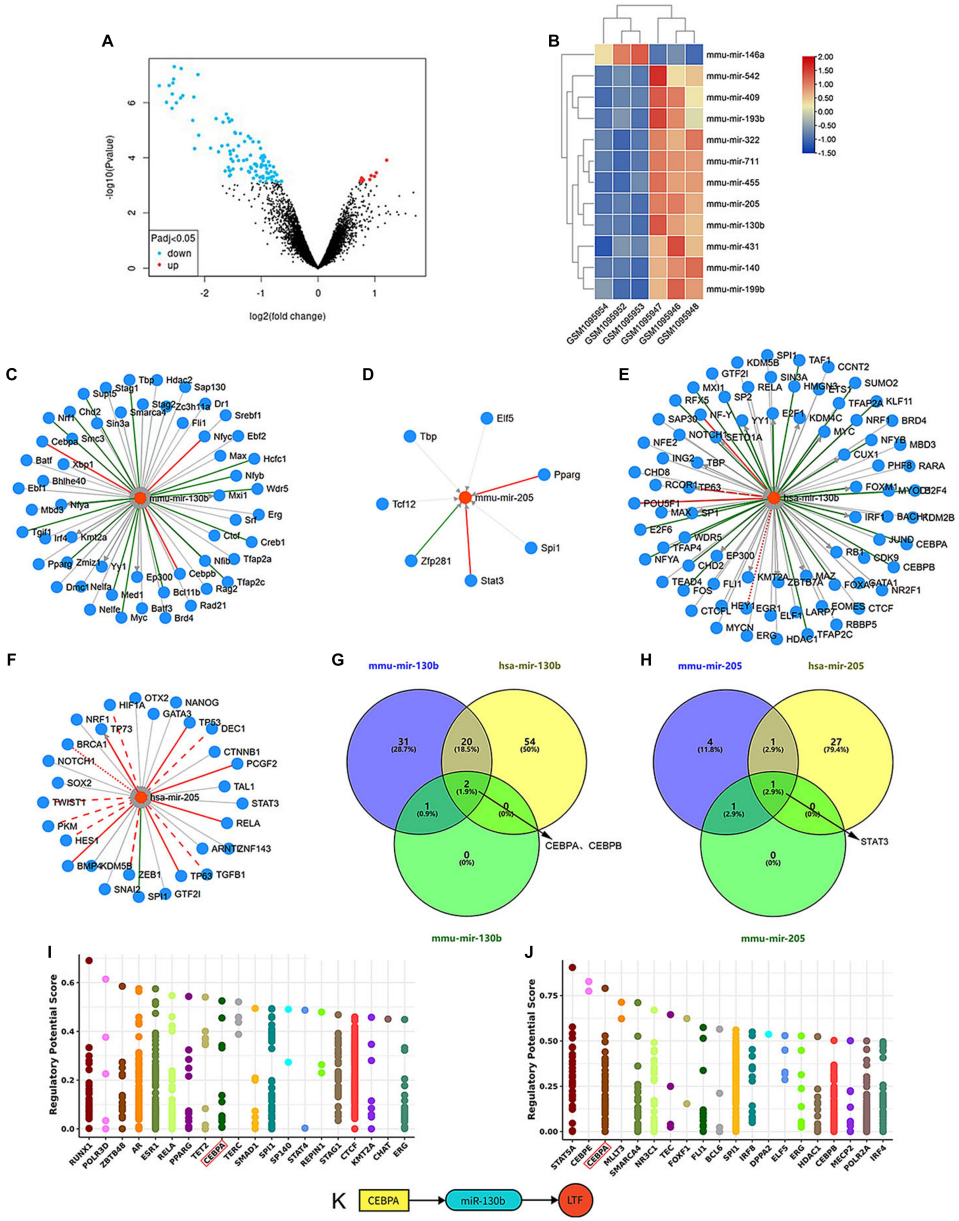
TF-miRNA-mRNA network in cochlear ferroptosis. **(A)** The volcano diagram of GSE45026 shows 7512 DEMs between the old group (GSM1095952~1095954) and young group (GSM1095946~1095948). **(B)** The heatmap shows 12 statistically significant DEMs. **(C–F)** TFs predicted by mmu-mir-130b **(C)**, mmu-mir-205 **(D)**, hsa-mir-130b **(E)**, and hsa-mir-205 **(F)**. **(G)** CEBPA and CEBPB were predicted by mir-130b. **(H)** STAT3 was predicted by mir-205. **(I,J)** The top 20 TFs predicted by LTF in mice **(I)** and humans **(J)**, CEBPA (Red box) was identified through the intersection of **(I,J)**, and TFs predicted by miRNAs in **(G,H)**. **(K)** The regulatory network was constructed.

7512 DEMs between the old group (GSM1095952~1095954) and young group (GSM1095946~1095948) in the GSE45026 dataset (Data were shown in Supplementary material) were identified with GEO2R (Figure 3A). 12 of them were further identified by the indicators: |log2(FC)| > 1 and adjusted *p*-value <0.05 (Figure 3B).

### A potential TF-miRNA-mRNA network of ferroptosis in aging cochleae was constructed

LTF was highly homologous between mice and humans with a similarity of 76.53 (n) in the GeneCars database. Meanwhile, mmu-mir-130b and mmu-mir-205 from mouse miRNAs matched hsa-mir-130b and hsa-mir-205 from human miRNAs in the miRBase database.

TFs of NFYC, CEBPA, and CEBPB were predicted by mmu-mir-130b, and TFs of STAT3 and PPARG were predicted by mmu-mir-205 in the miRNet database. According to the TransmiR database, TFs predicted by mmu-mir-130b and mmu-mir-205 are shown in Figures 3C,D, and TFs predicted by hsa-mir-130b and hsa-mir-205 are shown in Figures 3E,F. These results show overlapping TFs of miR-130b are CEBPA and CEBPB, and the overlapping TF of miR-205 is STAT3 (Figures 3G,H).

The Cistrome DB database was used to predict TFs for LTF, and the top 20 TFs between mice and humans are shown in Figures 3I,J. CEBPA was the sole overlapping TF by combining the above methods of predicting TFs. Therefore, it was hypothesized that the TF-miRNA-mRNA network of cochlear ferroptosis regulated by LTF between mice and humans was CEBPA-miR-130b-LTF (Figure 3K).

### Ferroptosis exists in aging HEI-OC1 cells with decreased LTF

**Figure 4:**
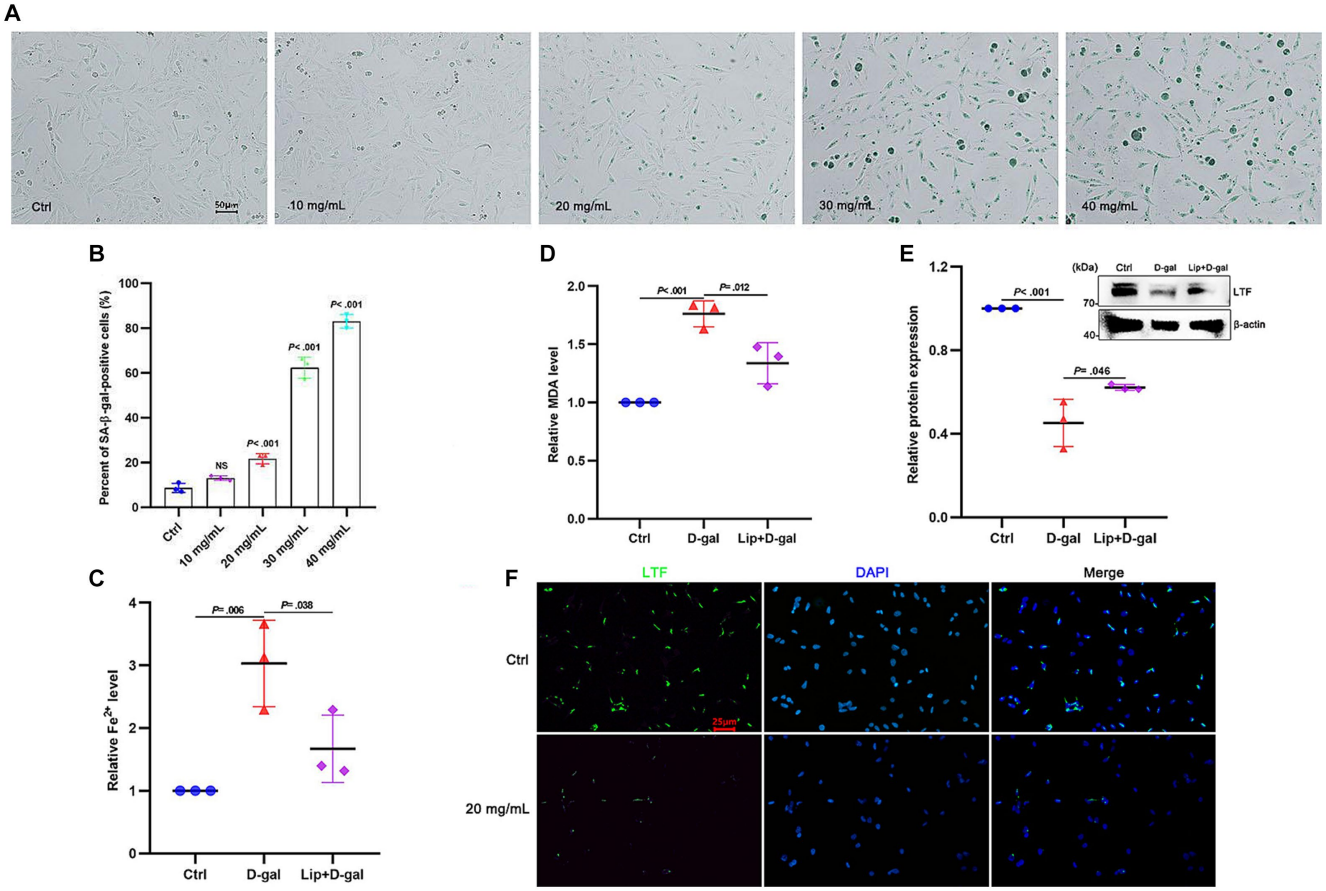
Ferroptosis in aging HEI-OC1 cells. **(A)** SA-β-gal staining in HEI-OC1 cells treated with different concentrations of D-gal for 48 h. **(B)** Quantification of SA-β-positive cells in **(A)**. Compared with the control group, the percentage of positive cells is observed to have a statistical difference at 20 mg/mL D-gal (*p* < 0.001; *N* = 3). **(C–E)** Compared with the control group, the expression of Fe^2+^ **(C)** and MDA **(D)** is increased in aging HEI-OC1 cells with 20 mg/mL D-gal (*p* = 0.006 and *p* < 0.001; *N* = 3). Oppositely, the expression of LTF **(E)** is decreased (*p* < 0.001; *N* = 3). More importantly, Liproxstatin-1 reversed the above phenomenon of ferroptosis in aging HEI-OC1 cells with 20 mg/mL D-gal (*p* = 0.038, *p* = 0.012, and *p* = 0.046; *N* = 3). **(F)** Immunofluorescence staining also shows decreased expression of LTF in aging HEI-OC1 cells with 20 mg/mL D-gal (LTF-green, DAPI-blue). Ctrl, control; Lip, Liproxstatin-1.

D-galactose (D-gal) has been widely used to induce senescence in various models (Azman and Zakaria, 2019), and the HEI-OC1 auditory cell line has been commonly used to investigate functions and mechanisms of hair cells *in vitro* (Zhang et al., 2021; Nan et al., 2022). To verify the occurrence of ferroptosis in aging HEI-OC1 cells, different concentrations of D-gal were applied to the HEI-OC1 cells. Compared with the control group, β-galactosidase activity was increased in the HEI-OC1 cells in a dose-dependent manner (Figure 4A). At a concentration of 20 mg/mL, the extent of senescence of the HEI-OC1 cells was significantly different from the control group (*p* < 0.001; Figure 4B). Therefore, 20 mg/mL D-gal was used for subsequent experiments. The results showed that the expression of Fe^2+^ and MDA were increased (*p* = 0.006 and *p* < 0.001; Figures 4C,D), while the expression of LTF was decreased (*p* < 0.001; Figure 4E) in aging HEI-OC1 cells. To better demonstrate that ferroptosis exists in aging HEI-OC1 cells, 5 μm Liproxstatin-1 (Zheng et al., 2020) was applied to HEI-OC1 cells with D-gal for 48 h. As expected, Liproxstatin-1 (ferroptosis inhibitor) reversed the above phenomenon of ferroptosis in aging HEI-OC1 cells (*p* = 0.038, *p* = 0.012, and *p* = 0.046; Figures 4C–E). Meanwhile, the fluorescence intensity of LTF was also decreased after the induction of D-gal (Figure 4F).

### Ferroptosis is certificated in aging cochlear explants with low expression of LTF

**Figure 5:**
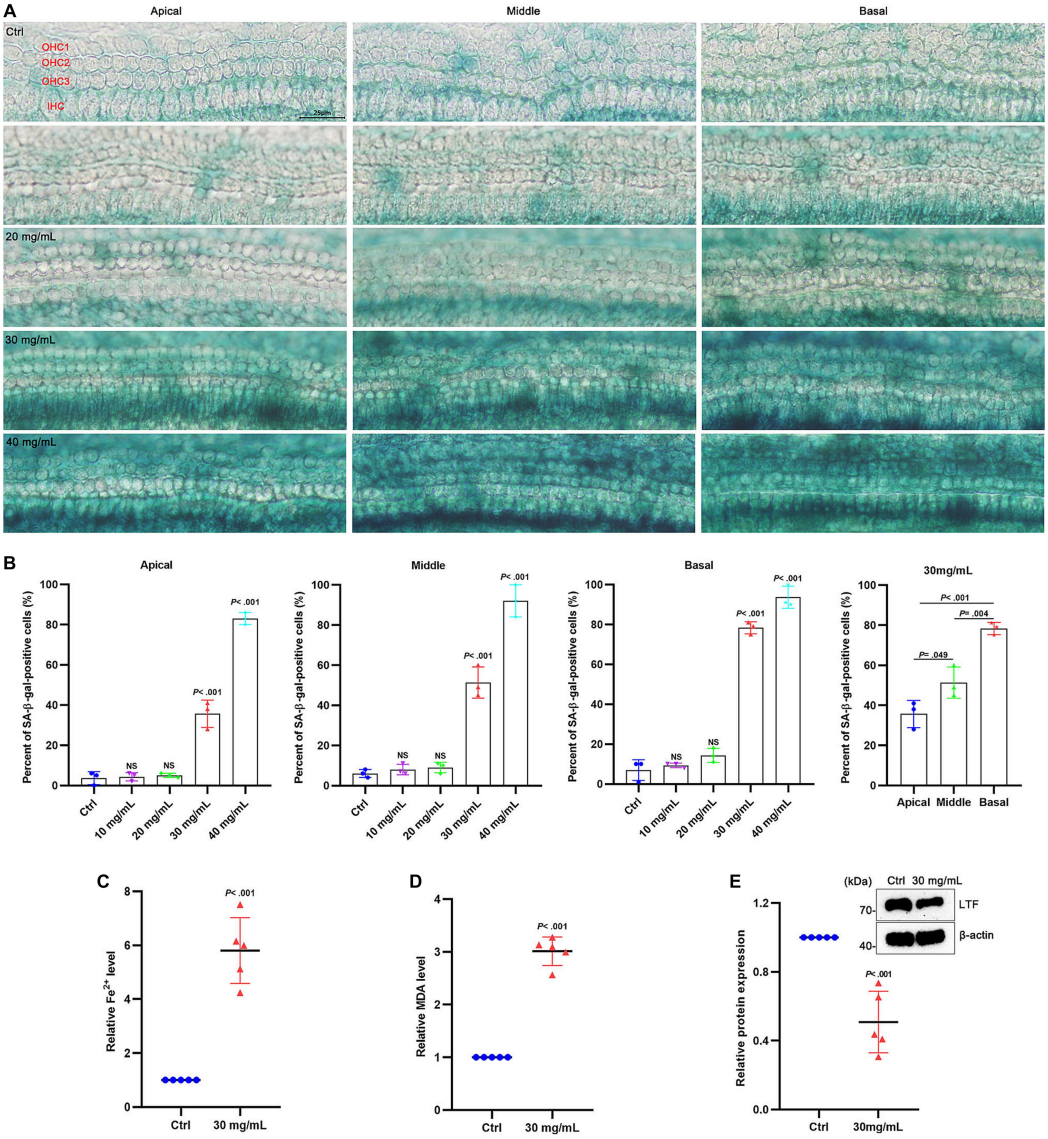
Ferroptosis in aging cochlear explants. **(A)** SA-β-gal staining in the basement membrane treated with different concentrations of D-gal for 48 h. **(B)** Quantification of SA-β-positive cells in **(A)**. Compared with the control group, the percentage of positive cells is observed to have a statistical difference at 30 mg/mL D-gal (*p* < 0.001; *N* = 3). **(C–E)** Compared with the control group, the expression of Fe^2+^ **(C)** and MDA **(D)** is increased in aging cochlear explants with 30 mg/mL D-gal (*p* < 0.001; *N* = 5). Oppositely, the expression of LTF **(E)** is decreased in aging cochlear explants with 30 mg/mL D-gal (*p* < 0.001; *N* = 5). OHC, outer hair cell; IHC, inner hair cell; Ctrl, control.

To verify whether ferroptosis occurred in aging cochlear explants, D-gal of gradient concentration was applied to the cochlear explants for 48 h. The hair cells in the basilar membrane exhibited obvious senescence after applying D-gal, with increased β-galactosidase activity at the base of the cochleae than at the apex (Figure 5A). The extent of senescence was also dose-dependent (Figure 5B). At a concentration of 30 mg/mL D-gal, the extent of senescence was significantly different from the control group (*p* < 0.001; Figure 5B). Therefore, 30 mg/mL was used for the later experiments. After 30 mg/mL D-gal induction, the expression of Fe^2+^ (Figure 5C) and MDA (Figure 5D) was increased more than 5 times and about 3 times, respectively, compared with the control group (All *p* < 0.001), while the expression of LTF was significantly decreased (*p* < 0.001; Figure 5E). These results indicate the occurrence of ferroptosis in aging cochlear explants.

### Aging mouse exhibits ferroptosis in cochleae with descending LTF

**Figure 6:**
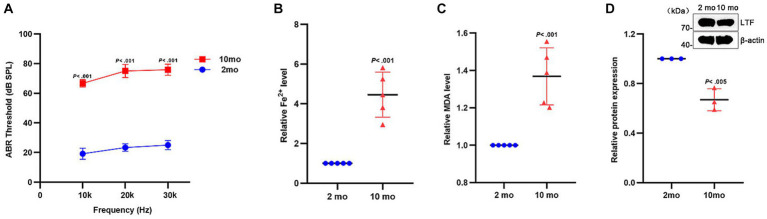
Ferroptosis in aging cochleae. **(A)** Compared with young mice (2mo), the distinctly increased ABR thresholds are shown in old mice (10mo) at all frequencies (*p* < 0.001; *N* = 6 mice). **(B–D)** Compared with young mice, the expression of Fe^2+^ **(B)** and MDA **(C)** is increased in aging cochleae (*p* < 0.001; *N* = 5). Oppositely, the expression of LTF **(D)** is decreased (*p* < 0.005; *N* = 3).

C57BL/6J mice, a predominant animal model for the study of ARHL, with high-frequency hearing loss starting at 3 months and gradually exhibiting hearing loss at full-frequency over time (Bowl and Dawson, 2019) To further verify the occurrence of ferroptosis in the aging cochleae, 2-month and 10-month-old C57BL/6J mice were selected for comparative analysis. As shown in Figure 6A, the mice developed severe hearing loss at 10 months (*p* < 0.001). As shown in Figures 6B,C, the expression of Fe^2+^ and MDA were both increased in the old group (All *p* < 0.001). Similarly, LTF expression was decreased in the aging cochleae (*p* < 0.005; Figure 6D). These results strongly suggest that ferroptosis occurs in the aging cochleae.

## Discussion

ARHL is a global problem, affecting approximately one third of people over 65 years of age worldwide (Wang and Puel, 2020). Despite massive investments in ARHL research, there is still no ideal prevention or treatment. Thus, it is crucial to find novel therapeutic targets for ARHL. In this study, the relationship between ARHL and ferroptosis was identified by bioinformatics, hub gene LTF was screened out and its potential regulatory mechanism was established. Finally, the results of bioinformatic analysis were preliminarily verified by experiments *in vitro* and *in vivo*. Our results show that LTF is a hub gene for regulating cochlear ferroptosis which provides important evidence for the treatment of ARHL and lays the foundation for the later verification of molecular mechanism.

Unlike necrosis or apoptosis, ferroptosis is an iron-dependent cell death pattern driven by lipid peroxidation accumulation (Dixon et al., 2012). Iron-dependent Fenton reaction is essential for ferroptosis and reducing lipid peroxidation can effectively inhibit ferroptosis (Jiang et al., 2021). As a pivotal actor in maintaining iron homeostasis (Rosa et al., 2017), LTF has been proven to prevent the Fenton reaction by sequestering Fe^3+^ (Superti, 2020) and positively regulating lipid metabolism (Xiong et al., 2018). Recently studies have shown that LTF plays an important role in age-related neurodegenerative diseases (Li B. et al., 2022) and relieves neuronal ferroptosis in intracerebral hemorrhagic stroke (Xiao et al., 2022). Moreover, CEBPA is a key transcription factor in adipogenesis (Riera-Heredia et al., 2022), and miR-130b is a post-transcriptional regulator of lipid metabolism (Luo et al., 2022). MiR-130b-3p, a mature sequence of miR-130b, has been confirmed to prevent ferroptosis by reducing iron accumulation and lipid peroxidation (Liao et al., 2021). These studies suggested that LTF, CEBPA, and miR-130b may jointly affect ferroptosis by regulating lipid peroxidation.

Consistent with the above theory, LTF, the only hub gene of ferroptosis in the mouse cochleae, was found to be highly homologous between mice and humans using bioinformatics in this work. Furthermore, CEBPA and miR-130b were confirmed to be TF and miRNA, respectively, that regulate LTF expression in the above two species. This suggests that CEBPA-miRNA-130B-LTF (TF-miRNA-mRNA) may be a potential regulatory network that regulates cochlear ferroptosis. Despite the lack of gene data from the cochlear tissue of ARHL patients, the regulatory network we constructed in this work is highly applicable to mice and human species. This will provide a new theoretical basis for regulating ARHL and the feasibility of animal experiments and clinical studies in the future.

As anticipated, aging HEI-OC1 cells and aging cochlear explants both showed pathological changes of ferroptosis which includes Fe^2+^ overload, lipid peroxidation, and low expression of LTF. Notably, the senescence of hair cells in the basilar membrane exhibited a tonotopic gradient and a concentration-dependent change, which is consistent with the pathological characteristics of ARHL (Wang and Puel, 2020). Moreover, this data shows that C57BL/6J mice suffered severe hearing loss at 10 months, with the occurrence of both iron overload, lipid peroxidation, and decreased expression of LTF. The above results prove the existence of cochlear ferroptosis in ARHL and LTF may be a hub gene via ferroptosis in ARHL progression.

More importantly, low-density lipoprotein receptor-related protein 1 (LRP1) was found to localize in the blood-labyrinth barrier and inner hair cells according to new research (Shi et al., 2022). As an important receptor for LTF (Li and Guo, 2021), LRP1 has the potential to break the restriction of BLB and deliver LTF to the inner ear. In combination with this theory, we will conduct experiments to achieve the purpose of treating ARHL by regulating the expression of LTF in the inner ear. The limitation of this study is that regulatory mechanism has not been validated and RNA-silencing or gene editing technology should be used to achieve the target of functional verification *in vivo* or *in vitro*. Besides, the LTF expressions in the cochlear implant with immunofluorescent assays need to be further improved in the future.

In summary, LTF was identified as a hub gene of cochlear ferroptosis in ARHL and its associated TF-miRNA-mRNA regulation network was constructed. Our findings revealed the relationship between ferroptosis and ARHL and provided a potential therapeutic target for ARHL.

## Data availability statement

The datasets presented in this study can be found in online repositories. The names of the repository/repositories and accession number(s) can be found in the article/Supplementary material.

## Ethics statement

The animal study was approved by Animal Ethics Committee of Fujian Medical University (approval No. IACUC FJMU 2022-0623). The study was conducted in accordance with the local legislation and institutional requirements.

## Author contributions

CZ: Data curation, Methodology, Writing – original draft, Writing – review & editing. XG: Data curation, Methodology, Writing – review & editing. YC: Project administration, Software, Visualization, Writing – review & editing. YL: Writing – review & editing. JC: Funding acquisition, Resources, Writing – review & editing. ZC: Project administration, Software, Visualization, Writing – review & editing. CC: Project administration, Software, Visualization, Writing – review & editing. GY: Project administration, Software, Visualization, Writing – review & editing. CL: Formal analysis, Writing – review & editing.

## Footnotes

^1^ http://www.ncbi.nlm.nih.gov/geo

^2^ http://www.ncbi.nlm.nih.gov/geo/geo2r

^3^ http://www.webgestalt.org/

^4^ https://www.genecards.org/

^5^ https://bioinfogp.cnb.csic.es/tools/venny/index.html

^6^ http://www.string-db.org

^7^ http://www.targetscan.org/vert_72/

^8^ https://www.mirbase.org

^9^ https://www.mirnet.ca/miRNet/home.xhtml

^10^ http://www.cuilab.cn/

^11^ http://cistrome.org/db/#/
